# Supplementary material for: Energetic and durable all-polymer aqueous battery for sustainable, flexible power
Source: Nat Commun. 2024 Nov 5;15:9539. doi: 10.1038/s41467-024-53804-2 (PMC11535528; doi:10.1038/s41467-024-53804-2)
Supplement: Supplementary file 1 — Supplementary Information [file 41467_2024_53804_MOESM1_ESM.pdf]

Supporting information for

Energetic and durable all-polymer aqueous battery for sustainable, flexible power

Yang Hong[1,2], Kangkang Jia[1], Yueyu Zhang[3], Ziyuan Li[4], Junlin Jia[5], Jing Chen[6], Qimin Liang[1], Huarui Sun[1], Qiang Gao[6], Dong Zhou[7], Ruhong Li[8], Xiaoli Dong[9], Xiulin Fan[8]*, Sisi He[1]*

[1]School of Science, Harbin Institute of Technology (Shenzhen), Shenzhen 518055, China.

[2]Department of Chemistry and Biotechnology, School of Engineering, The University of Tokyo, Tokyo 113-8656, Japan.

[3]Wenzhou Institute University of Chinese Academy of Sciences, Wenzhou, 325001, China.

[4]School of Physical Science And Technology, Ningbo University, Ningbo, Zhejiang 315211, China.

[5]School of Physics, East China University of Science and Technology Shanghai 200237, China.

[6]School of Chemistry and Chemical Engineering, Yangzhou University, Yangzhou 225002, China.

[7]Tsinghua Shenzhen International Graduate School, Tsinghua University, Shenzhen 518055, China.

[8]State Key Laboratory of Silicon and Advanced Semiconductor Materials, School of Materials Science and Engineering, Zhejiang University, Hangzhou 310027, China.

[9]Department of Chemistry and Shanghai Key Laboratory of Molecular Catalysis and Innovative Materials, Institute of New Energy, iChEM (Collaborative Innovation Center of Chemistry for Energy Materials), Fudan University, Shanghai 200433, China.

These authors contributed equally: Yang Hong, Kangkang Jia.

Corresponding author.

email: xlfan@zju.edu.cn, hesisi@hit.edu.cn

Supplementary tables and figures

a

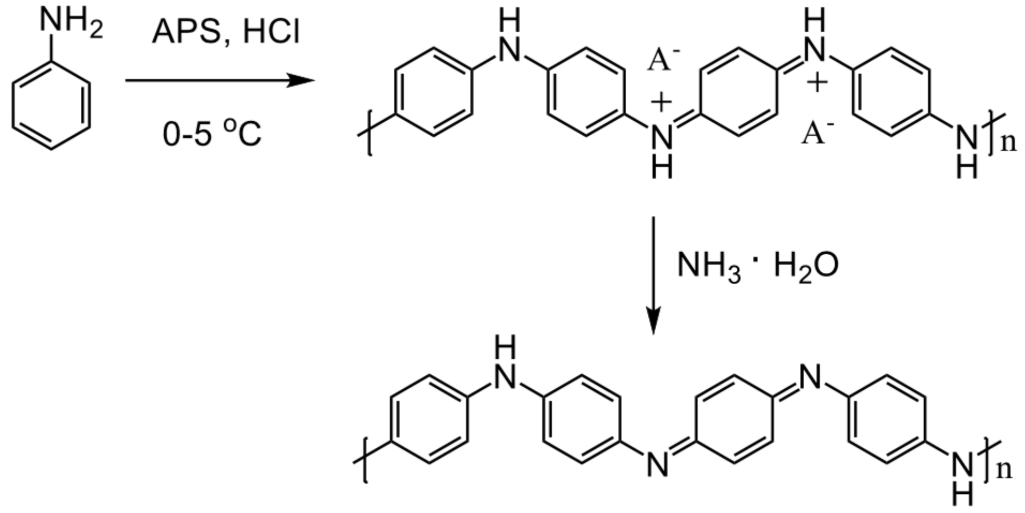

b

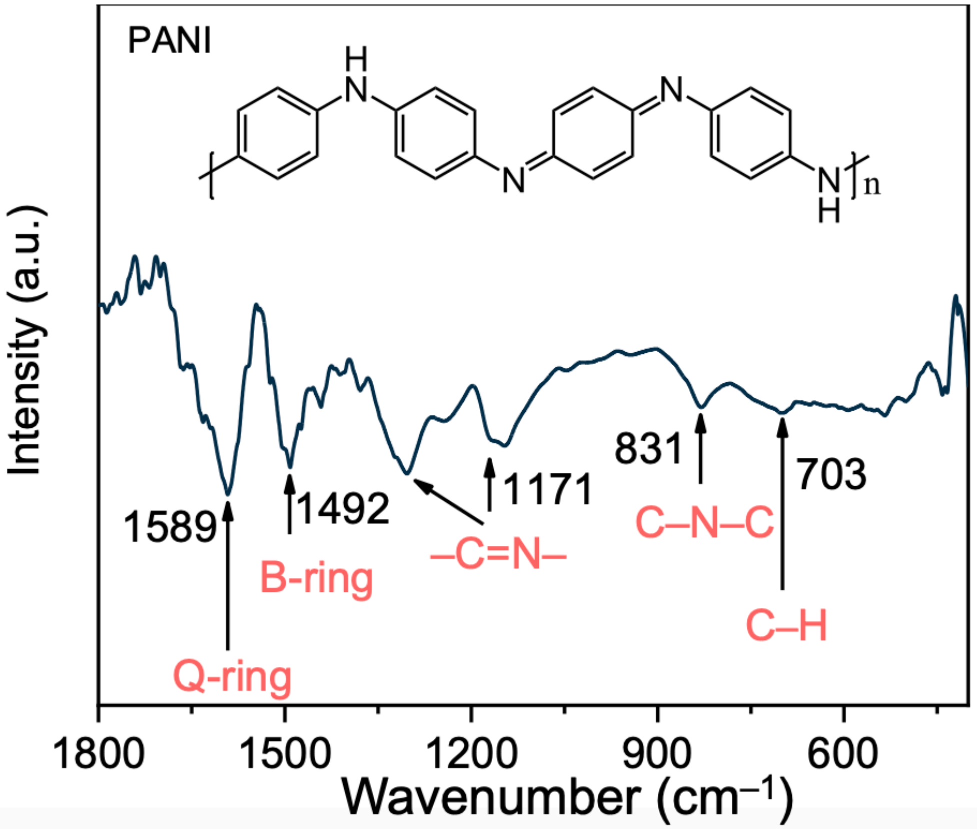

Supplementary Fig. 1. a, Synthesis route of PANI (emeraldine base). **b,** FTIR spectra of PANI (emeraldine base).

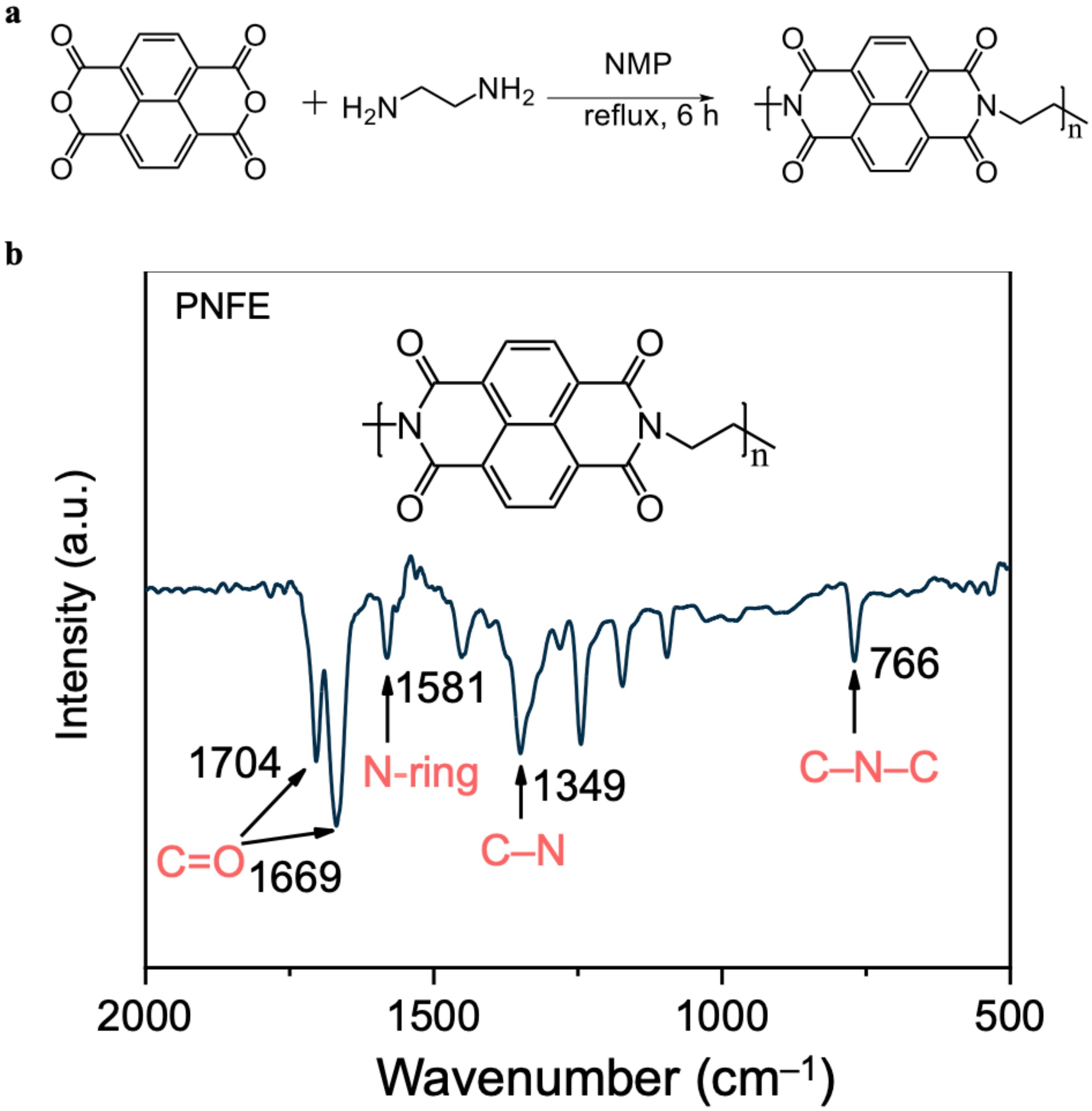

Supplementary Fig. 2. a, Synthesis route of PNFE. **b,** FTIR spectra of PNFE.

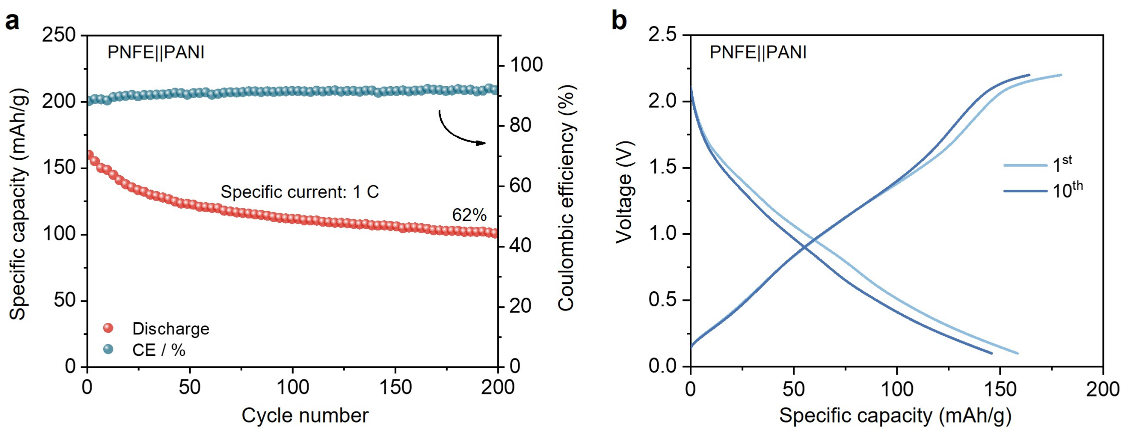

Supplementary Fig. 3. Electrochemical performances of the PNFE||PANI full battery. **a**, Cycling stability at a current density of 1 C (1 C=147 mA/g). **b**, Charge/discharge profiles in the first ten cycles at a current density of 1 C.

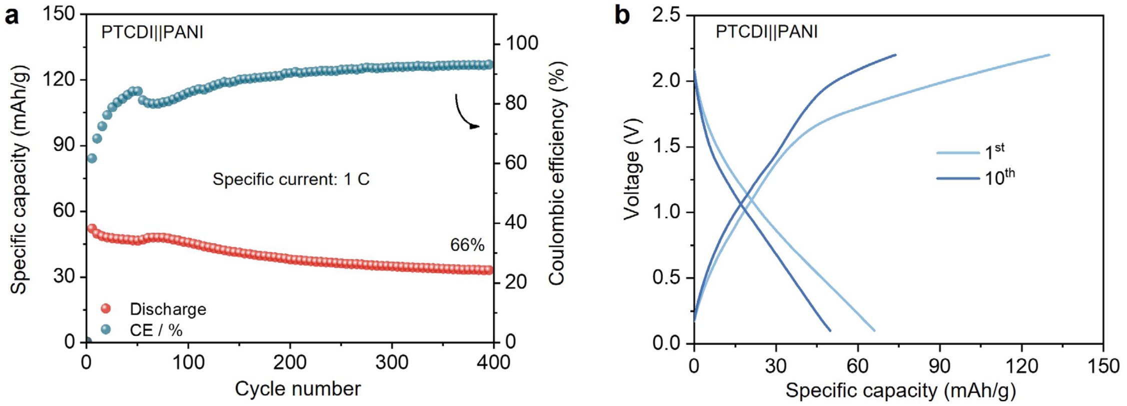

Supplementary Fig. 4. Electrochemical performances of the PTCDI||PANI full battery. **a**, Cycling stability at a current density of 1 C (1 C=147 mA/g). **b**, Charge/discharge profiles in the first ten cycles at a current density of 1 C.

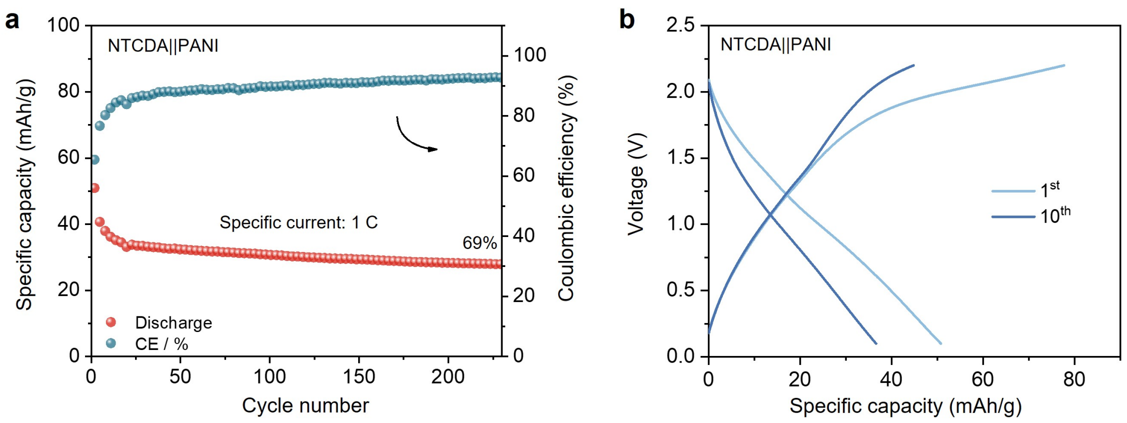

Supplementary Fig. 5. Electrochemical performances of the NTCDA||PANI full battery. **a**, Cycling stability at a current density of 1 C (1 C=147 mA/g). **b**, Charge/discharge profiles in the first ten cycles at a current density of 1 C.

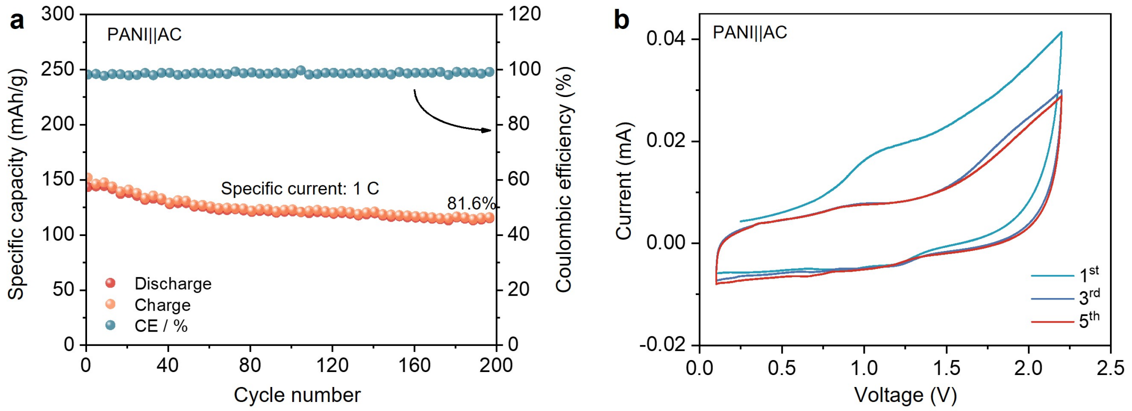

Supplementary Fig. 6. Electrochemical performances of the PANI||AC half battery. **a**, Cycling stability at a current density of 1 C (1 C=147 mA/g). **b**, CV curves of half battery at 1st, 3rd and 5th cycle.

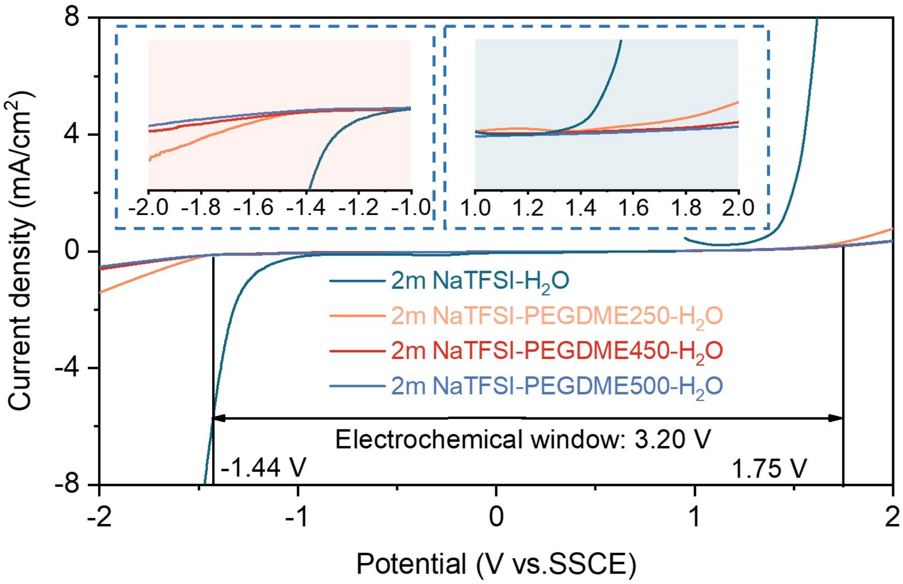

Supplementary Fig. 7. Electrochemical stability window of electrolytes, including 2m NaTFSI-94%PEGDME (250, 450, 500)-6%H_2O, and 2m NaTFSI-H_2O.

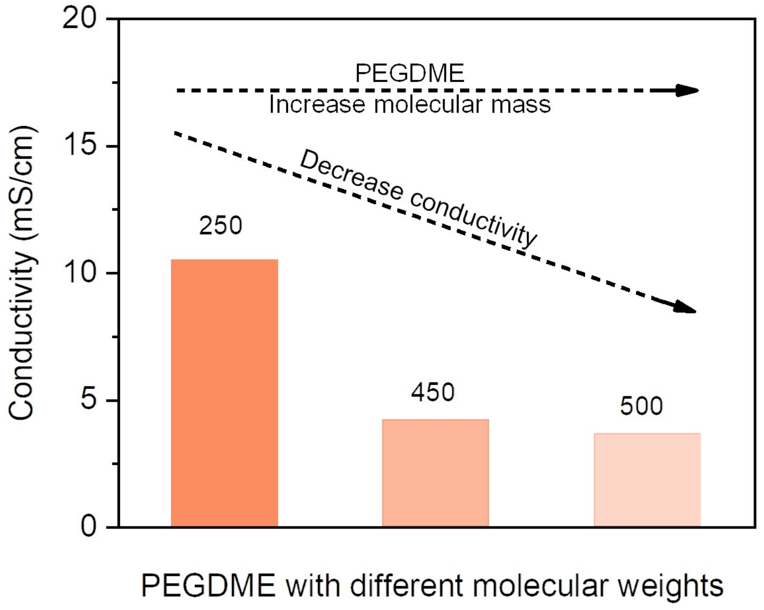

Supplementary Fig. 8. Ionic conductivity of 2m NaTFSI-94%PEGDME (250, 450, 500)-6%H_2O.

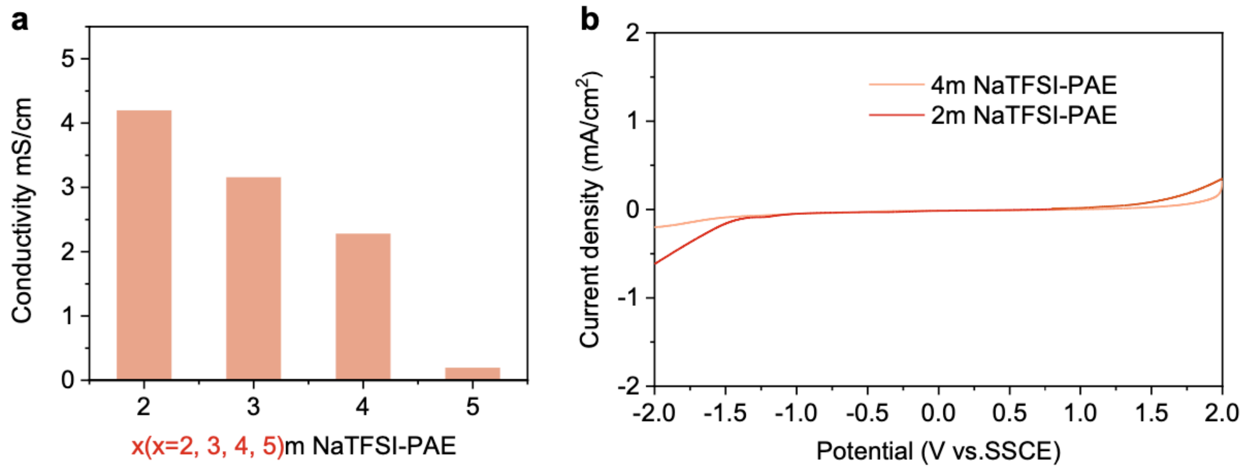

Supplementary Fig. 9. a, Ionic conductivities of 2m, 3m, 4m, and 5m NaTFSI-PAE. **b**, Electrochemical stabilities of 2m, and 4m NaTFSI-PAE.

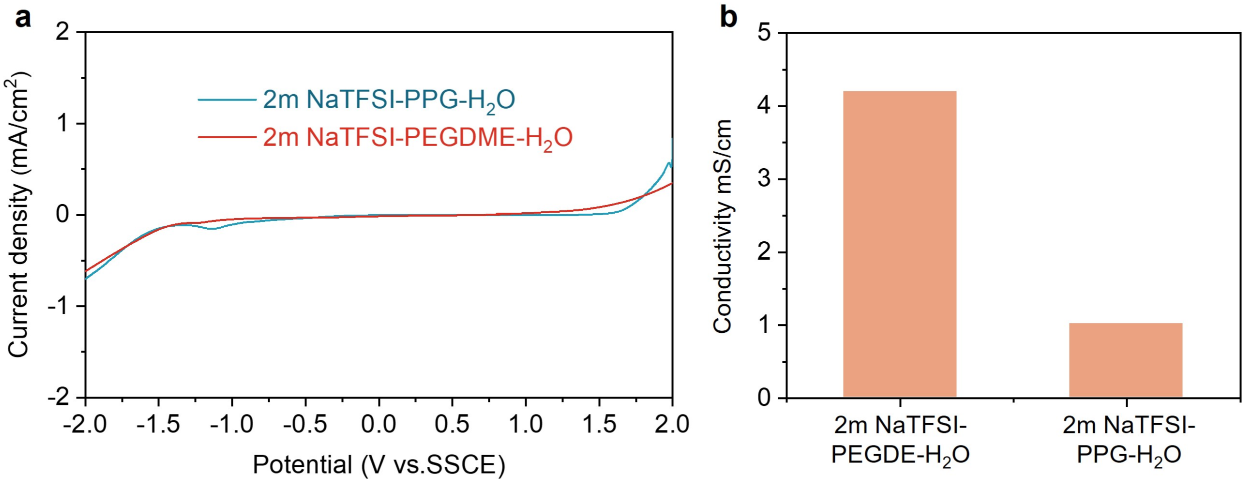

Supplementary Fig. 10. a, Electrochemical stabilities 2m NaTFSI-PEGDME-H_2O, and 2m NaTFSI-PPG-H_2O. **b,** Ionic conductivities of 2m NaTFSI-PEGDME-H_2O, and 2m NaTFSI-PPG-H_2O.

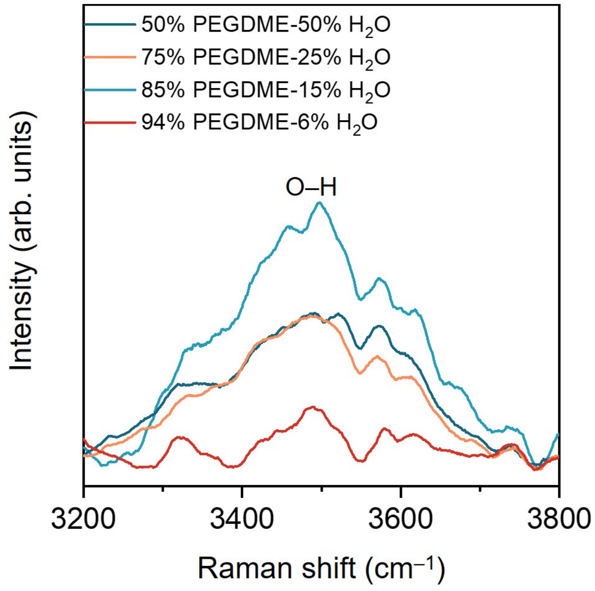

Supplementary Fig. 11. Raman spectra of electrolytes with different PEGDME concentrations (50, 75, 85 and 94% in weight).

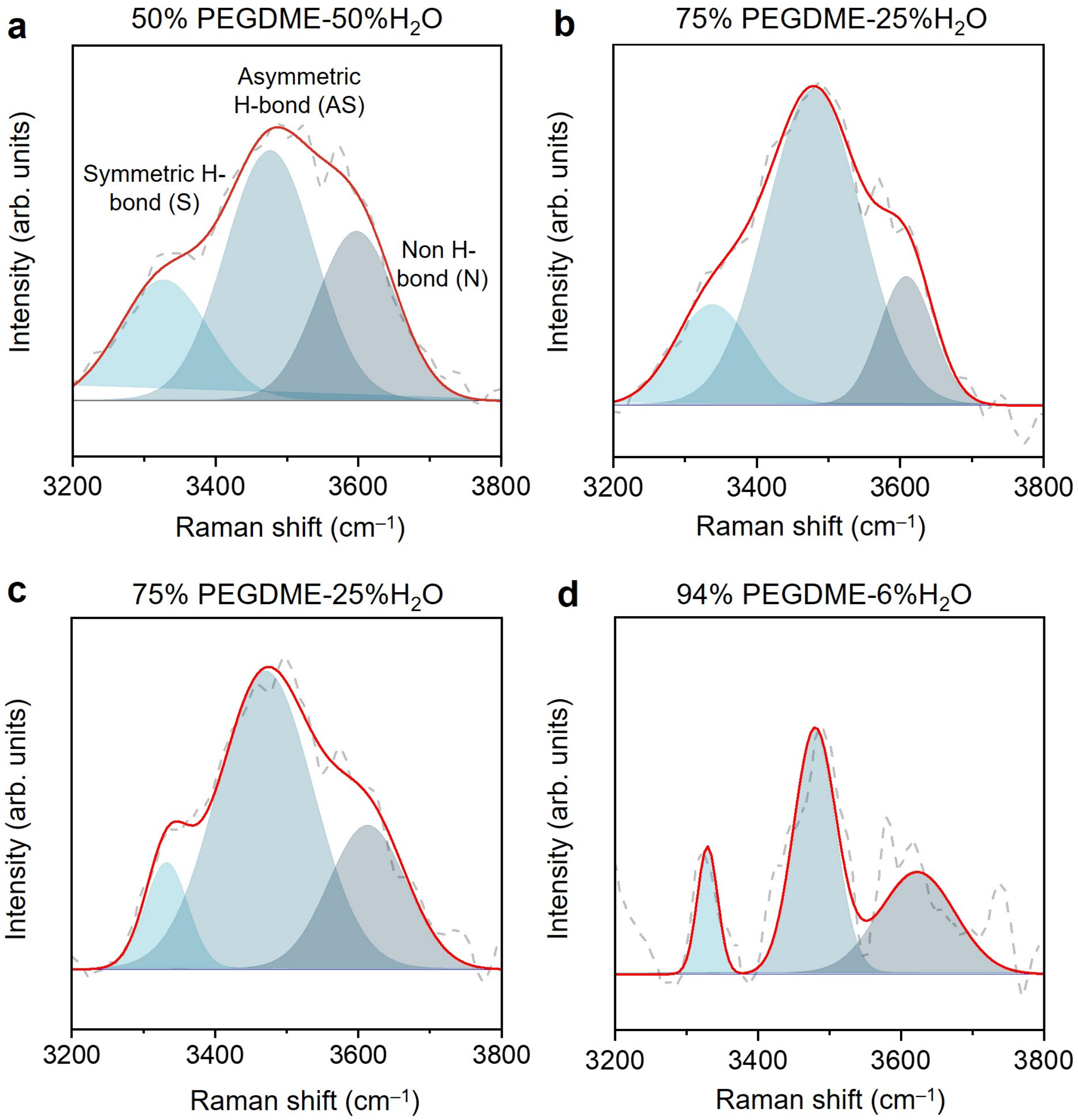

Supplementary Fig. 12. Peak fittings of the O–H stretching vibrations of water molecules with different hydrogen bonds (strong, weak, and non-hydrogen bonds). **a**, 50%PEGDME-50%H_2O. **b**, 75%PEGDME-25%H_2O. **c,** 85%PEGDME-15%H_2O. **d**, 94%PEGDME-6%H_2O.

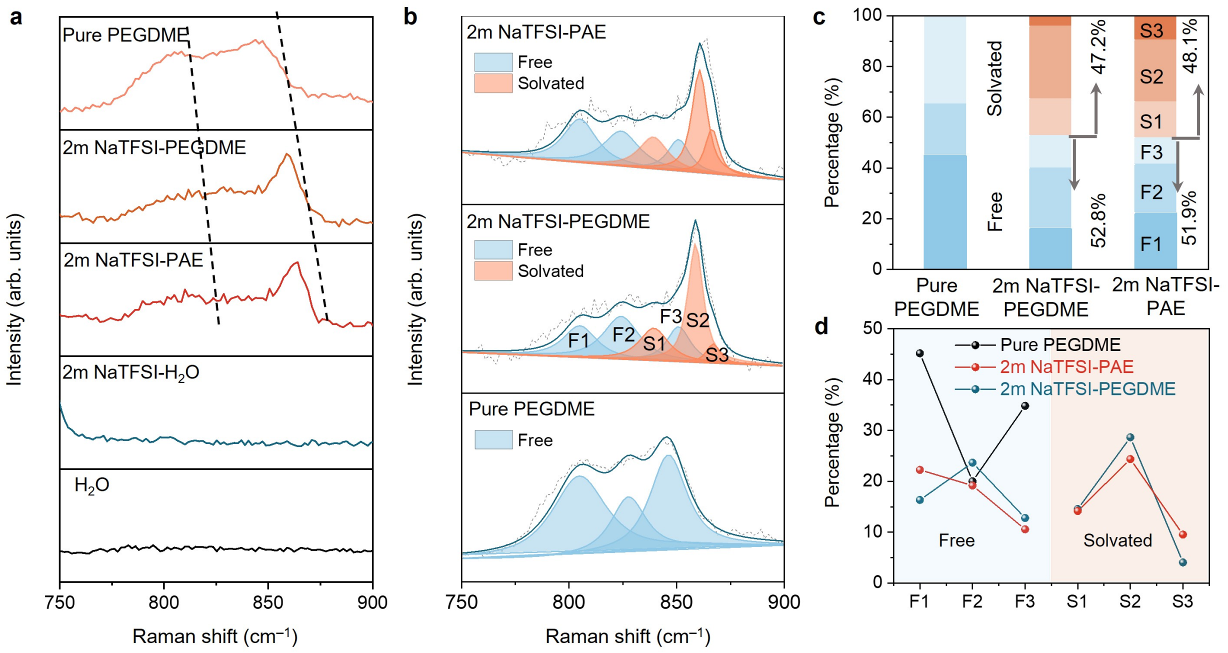

Supplementary Fig. 13. a, Raman spectra of pure PEGDME, 2m NaTFSI-PEGDME, 2m NaTFSI-PAE, 2m NaTFSI-H_2O, and H_2O. **b,** Fitting of Raman spectra of PEGDME, 2m NaTFSI-PEGDME, and 2m NaTFSI-PAE. **c, d** Ether ratio of PEGDME, 2m NaTFSI-PEGDME, and 2m NaTFSI-PAE solvation layers.

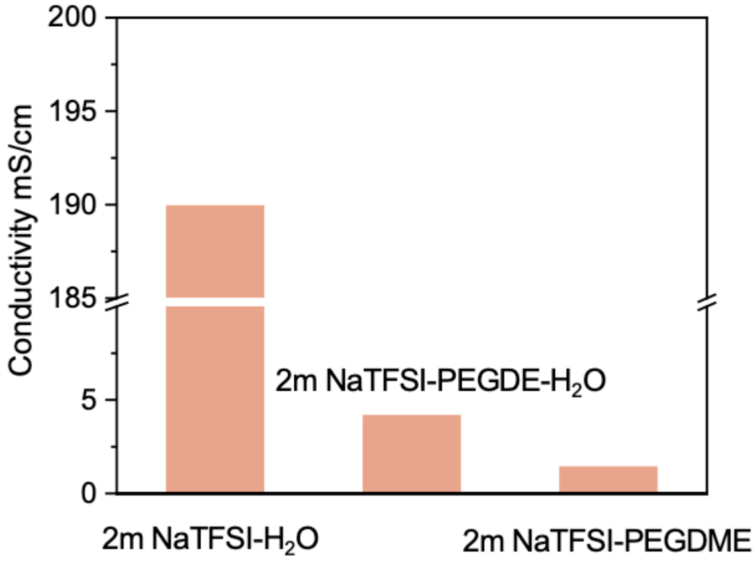

Supplementary Fig. 14. Ionic conductivities comparison of 2m NaTFSI-H_2O, 2m NaTFSI-PEGDME-H_2O, and 2m NaTFSI-PEGDME.

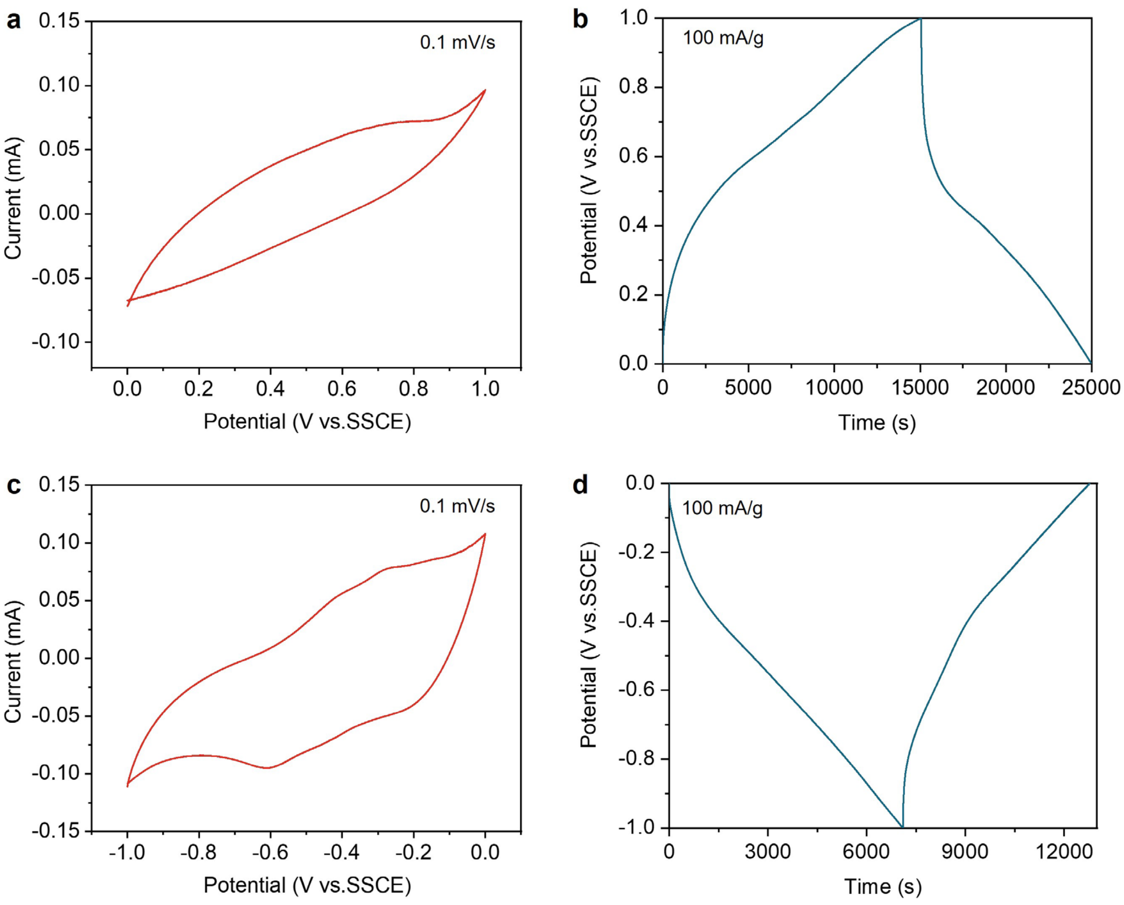

Supplementary Fig. 15. Electrochemical behavior of the PANI electrode in 2m NaTFSI polymer-aqueous electrolyte. **a**, CV test in 2m NaTFSI polymer-aqueous electrolyte at 0-1 V. **b**, Galvanostatic charge/discharge in 2m NaTFSI polymer-aqueous electrolyte at 0-1 V. **c**, CV test in 2m NaTFSI polymer-aqueous electrolyte at –1-0 V. **d**, Galvanostatic charge/discharge 2m NaTFSI polymer-aqueous electrolyte at –1-0 V.

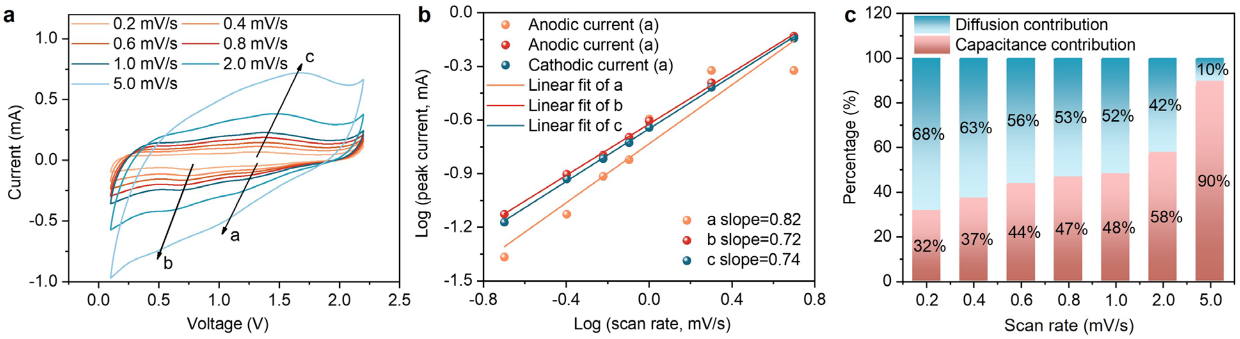

Supplementary Fig. 16. a, CV curves at different sweep rates (*v*) and **b**, corresponding log i_p versus log *v* of the PANI electrode (i_p= peak current). **c,** Ratio of diffusion and capacitance contributions at different scan rates.

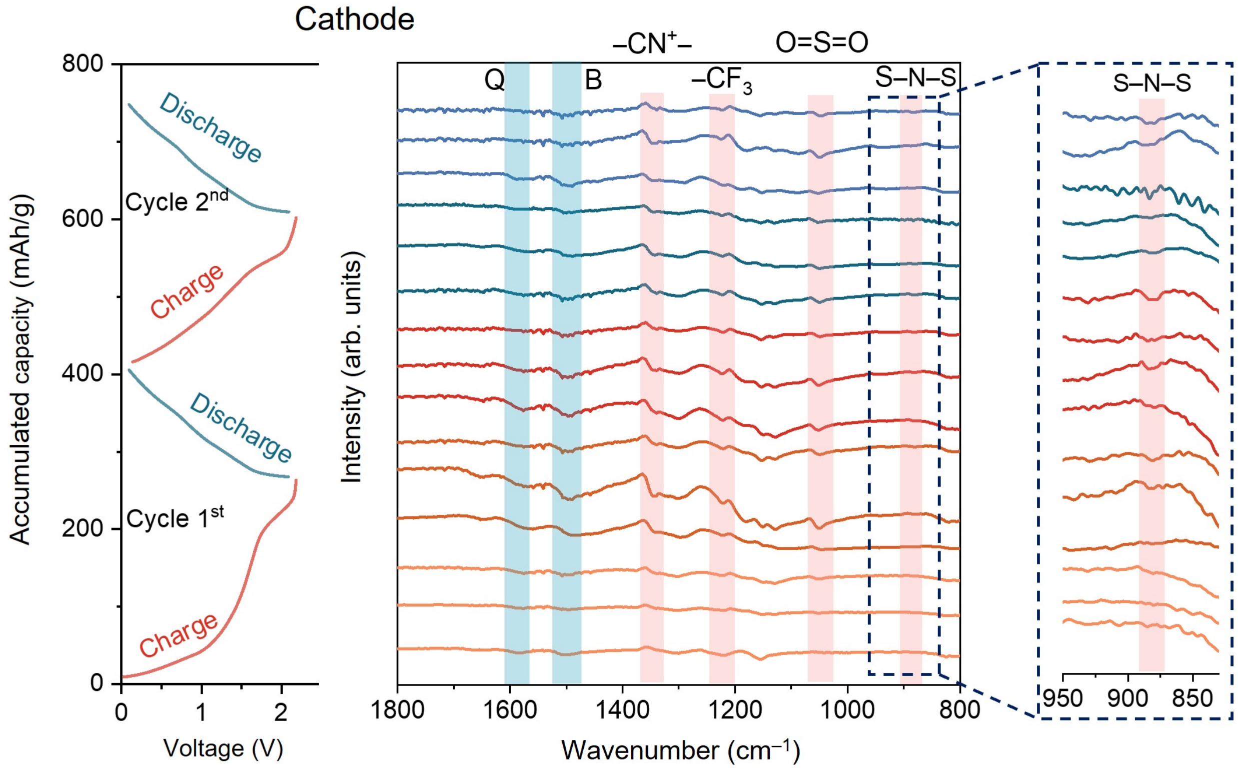

Supplementary Fig. 17. Actual FT–IR spectra of PANI cathode at different voltages in 1st, and 2nd cycles.

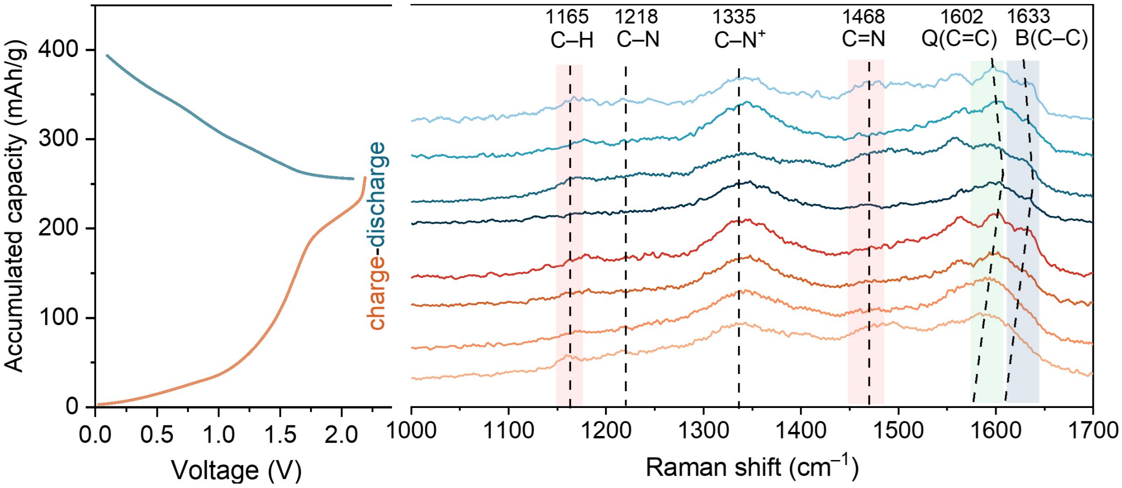

Supplementary Fig. 18. Raman spectra of PANI cathode at different voltage states.

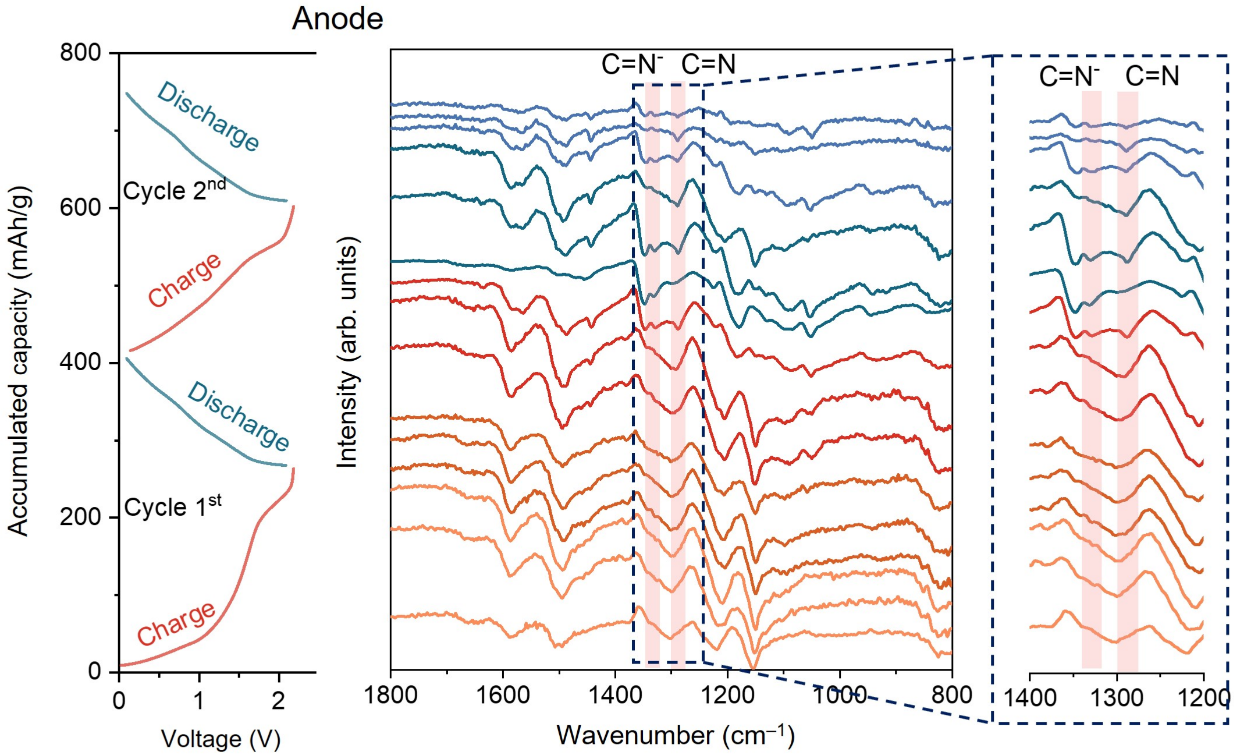

Supplementary Fig. 19. Actual FT–IR spectra of PANI anode at different voltages in 1st, and 2nd cycles.

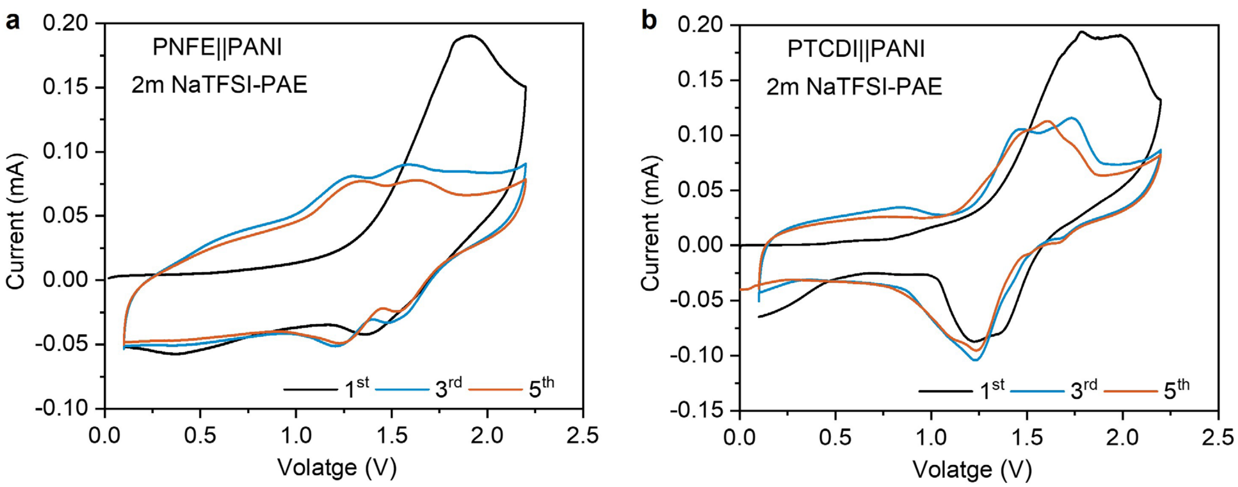

Supplementary Fig. 20. CV measurements with different organic electrodes in the 2m NaTFSI-PAE. a, PNFEI||PANI. b, PTCDI||PANI.

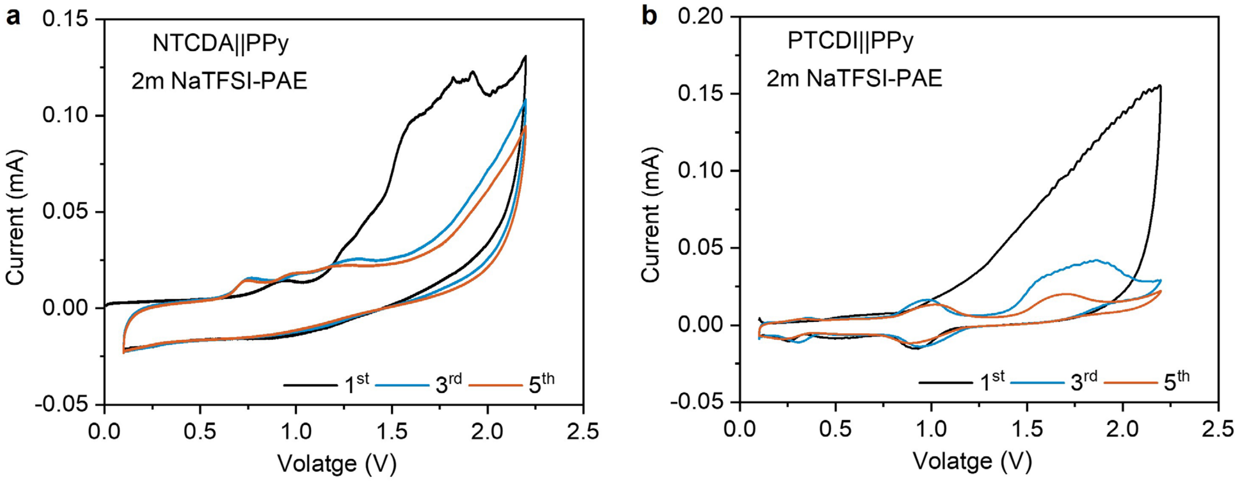

Supplementary Fig. 21. CV measurements with different organic electrodes in the 2m NaTFSI-PAE. a, NTCDA||PPy. b, PTCDI||PPy.

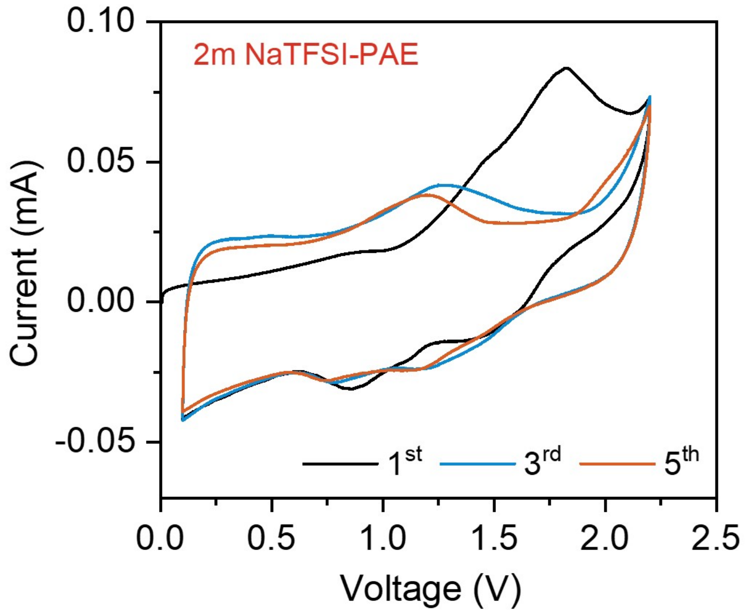

Supplementary Fig. 22. CV curves of all-PANI battery in 2m NaTFSI-PAE.

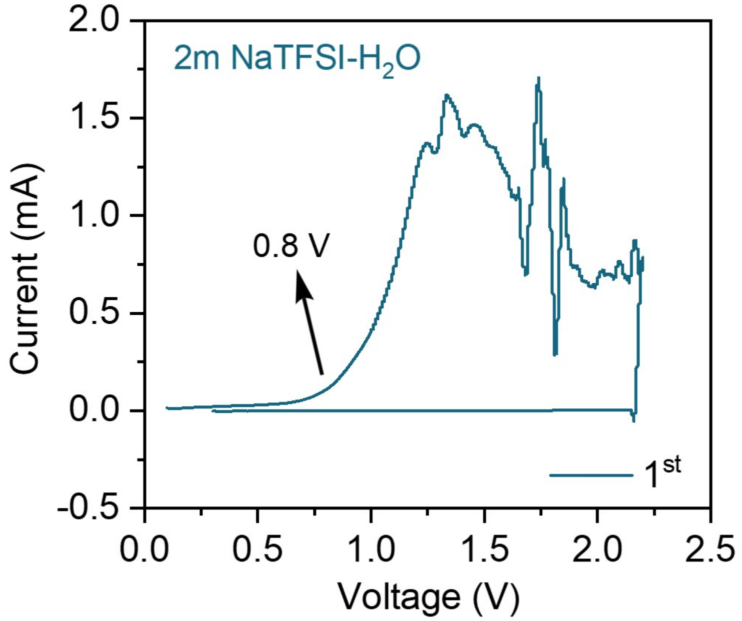

Supplementary Fig. 23. CV curves of all-PANI battery in 2m NaTFSI-H_2O.

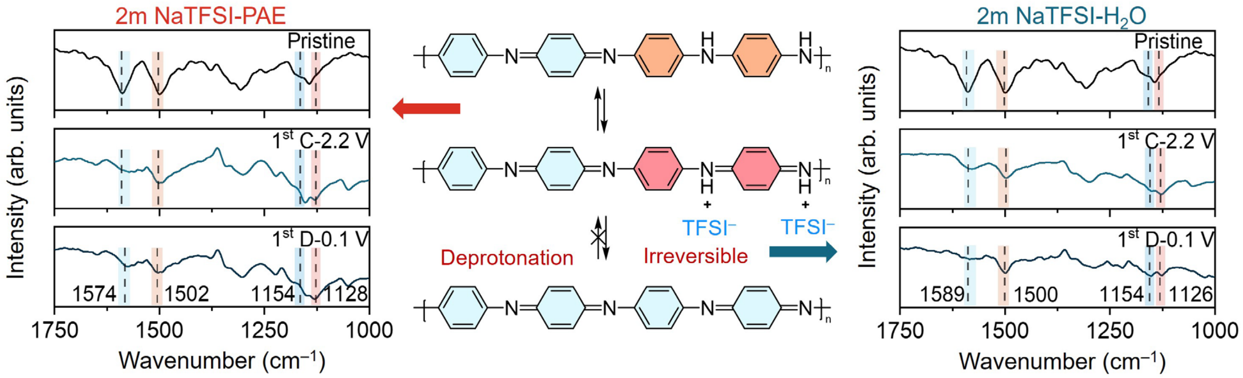

Supplementary Fig. 24. Different structure changes of polyaniline cathode during the discharge process in 2m NaTFSI-PAE, and 2m NaTFSI-H_2O, and corresponding FTIR spectra under different voltage (pristine, charged to 2.2 V, discharged to 0.1 V)

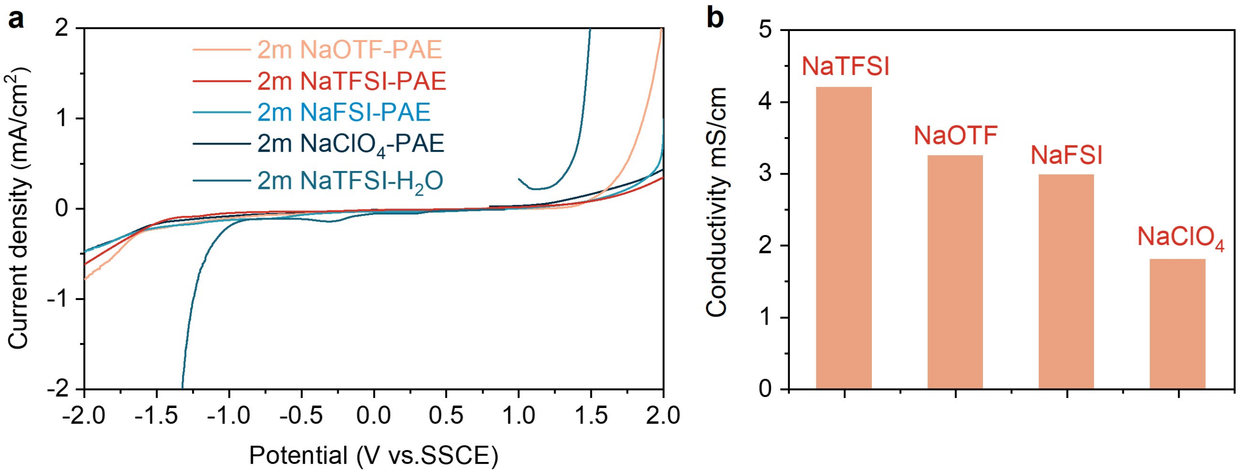

Supplementary Fig. 25. a, Electrochemical stabilities of 2m NaOTF-PAE, 2m $NaClO_4$-PAE, and 2m NaFSI-PAE, and 2m NaTFSI-H_2O. **b,** Ionic conductivities of 2m $NaClO_4$-PAE, 2m NaOTF-PAE, 2m NaFSI-PAE, and 2m NaTFSI-PAE.

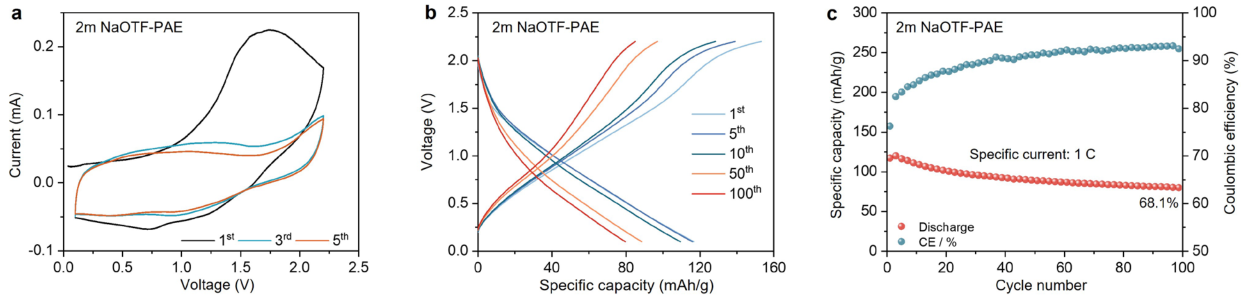

Supplementary Fig. 26. Electrochemical performances of all PANI batteries in 2m NaOTF-PAE. **a**, CV measurements. **b**, Cycling stability at a current density of 1 C (1 C=147 mA/g). **c**, Charge/discharge profiles in the first ten cycles at a current density of 1 C.

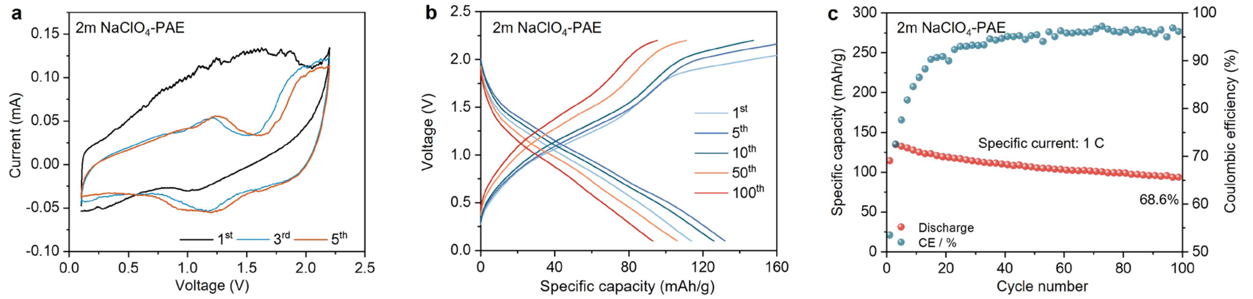

Supplementary Fig. 27. Electrochemical performances of all PANI batteries in 2m $NaClO_4$-PAE. **a**, CV measurements. **b**, Cycling stability at a current density of 1 C (1 C=147 mA/g). **c**, Charge/discharge profiles in the first ten cycles at a current density of 1 C.

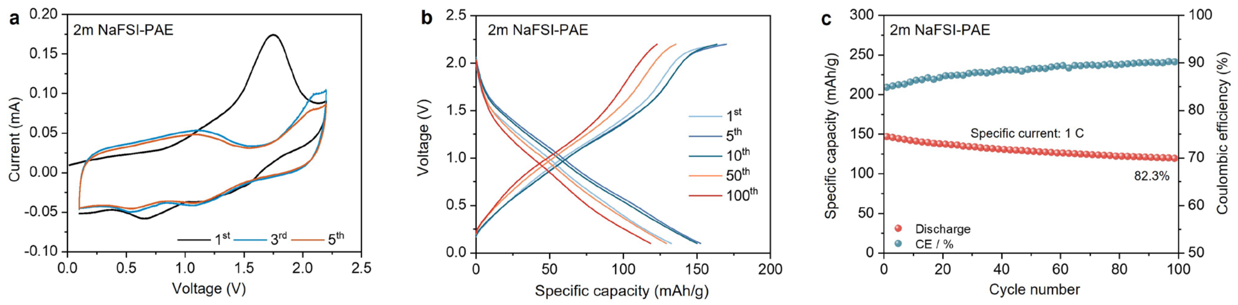

Supplementary Fig. 28. Electrochemical performances of all PANI batteries in 2m NaFSI-PAE. **a**, CV measurements. **b**, Cycling stability at a current density of 1 C (1 C=147 mA/g). **c**, Charge/discharge profiles in the first ten cycles at a current density of 1 C.

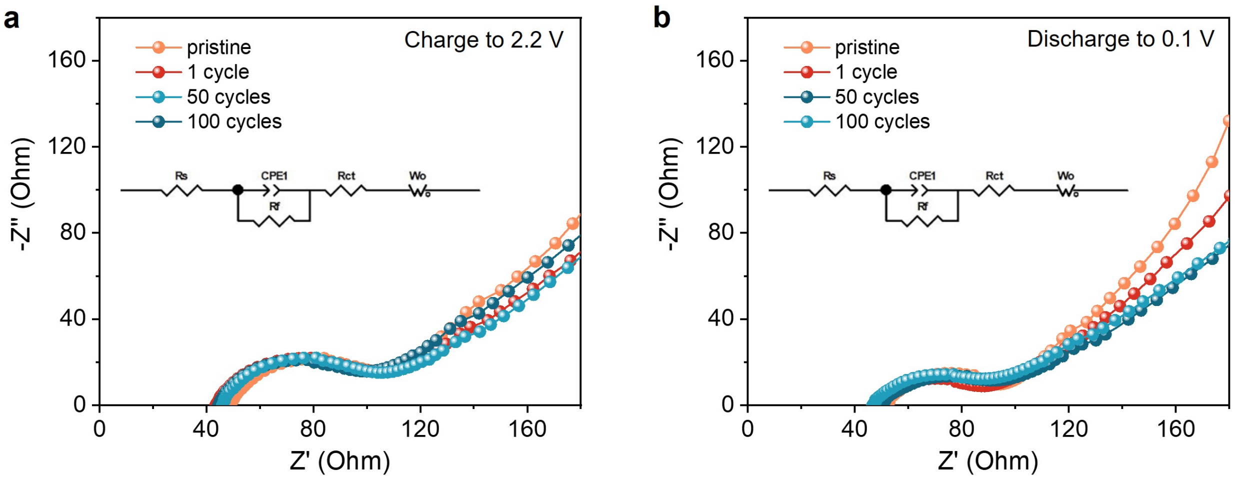

Supplementary Fig. 29. Nyquist plots of all-PANI battery charged to 2.2 V (**a**) and discharge to 0.1 V (**b**) after different cycles.

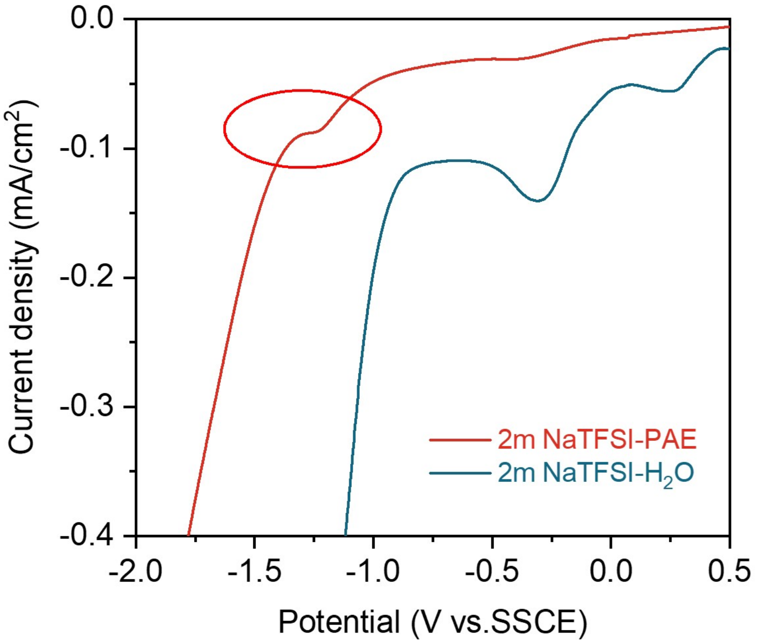

Supplementary Fig. 30. Magnified view of the LSV of the PAE in the –2.0-0.5 V voltage range (The red circled curve indicates the formation of SEI).

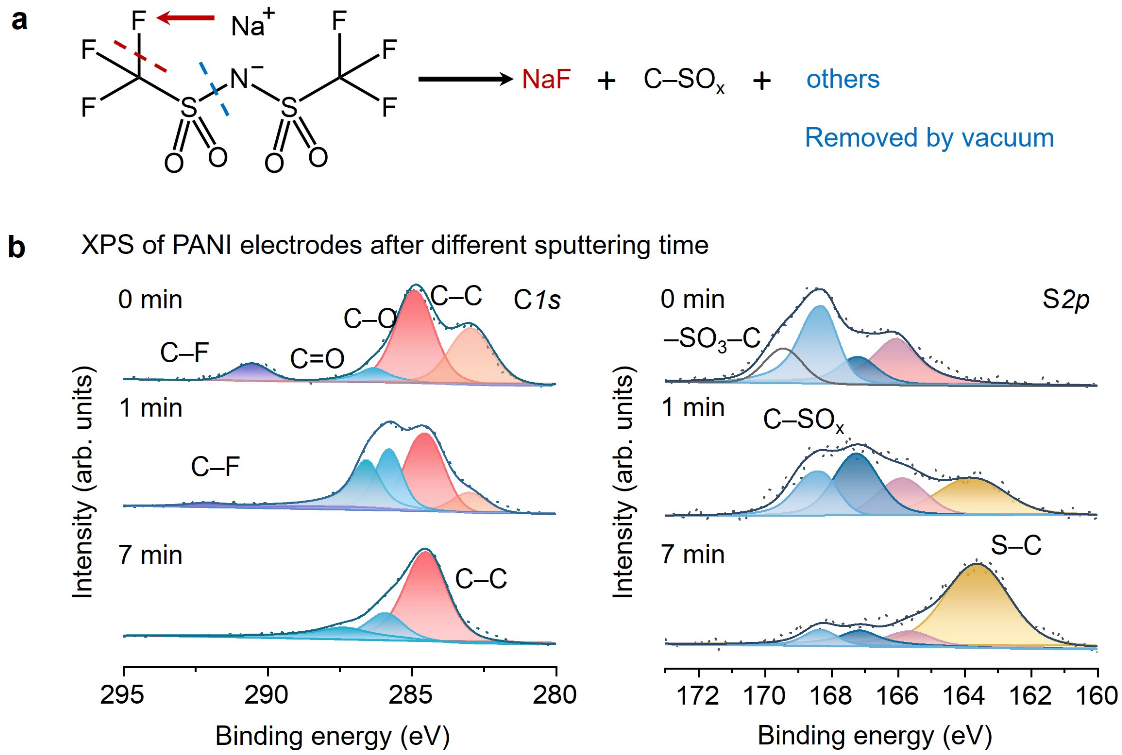

Supplementary Fig. 31. **a**, Decomposition reaction of NaTFSI on PANI anode surface. **b,** Fitting of XPS spectra of C*1s* and S*2p* after ion sputtering with 0, 1, and 7 min.

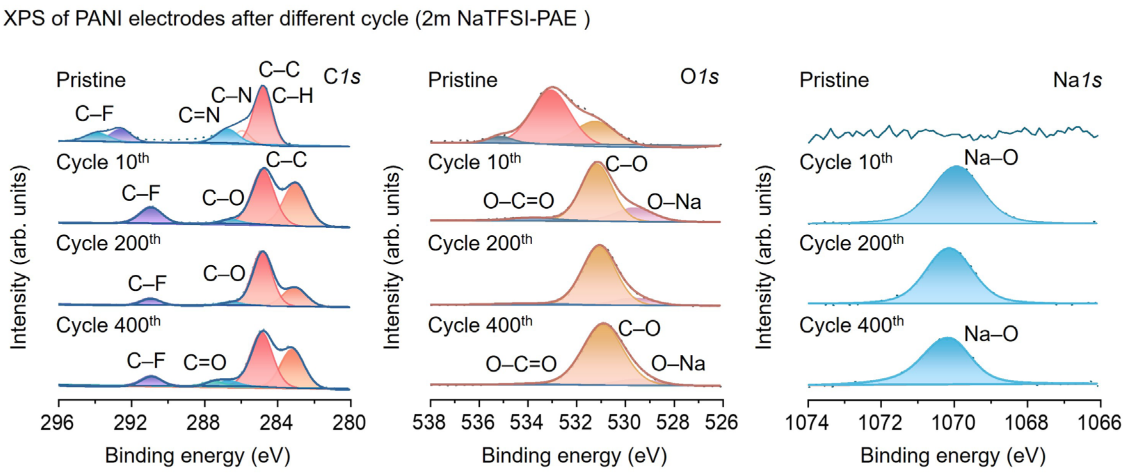

Supplementary Fig. 32. XPS spectra (C*1s*, O*1s*, Na*1s*) of SEI on polyaniline anode after different cycles (pristine, 10th, 200th, and 400th).

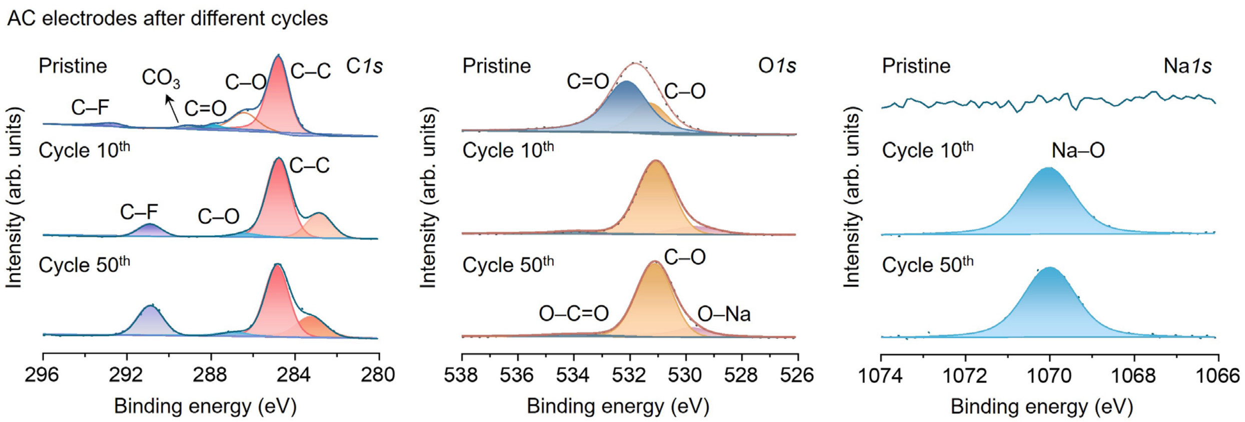

Supplementary Fig. 33. XPS spectra (C*1s*, O*1s*, and Na*1s*) of SEI on AC electrodes after different cycles (pristine, 10th, and 50th).

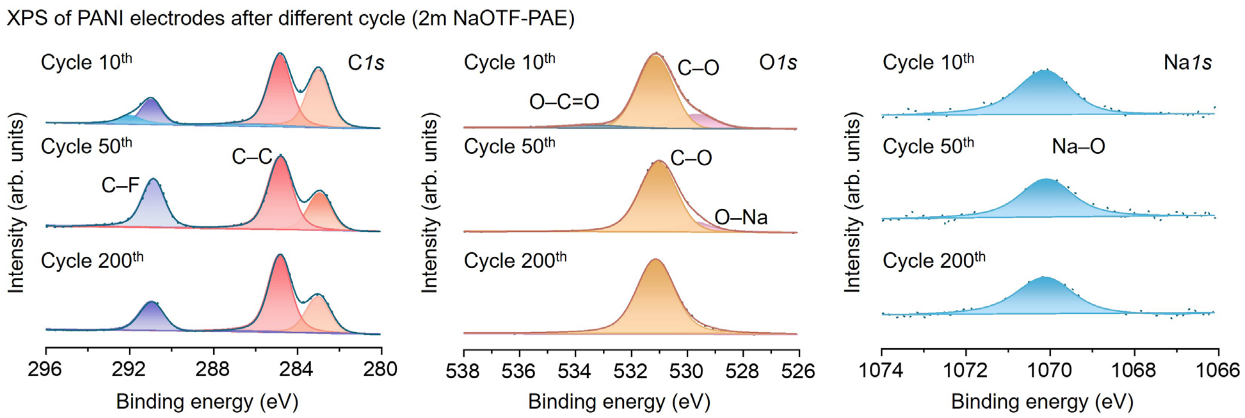

Supplementary Fig. 34. XPS spectra (C*1s*, O*1s*, and Na*1s*) of SEI on polyaniline anode after different cycles (10th, 50th, and 200th) in 2m NaOTF-PAE electrolyte.

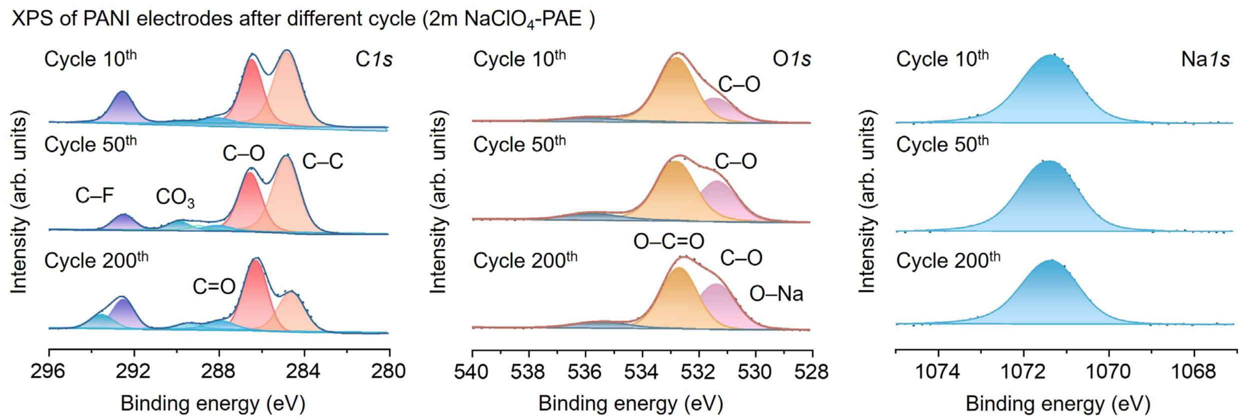

Supplementary Fig. 35. XPS spectra (C*1s*, O*1s*, and Na*1s*) of SEI on polyaniline anode after different cycles (10th, 50th, and 200th) in 2m $NaClO_4$-PAE electrolyte.

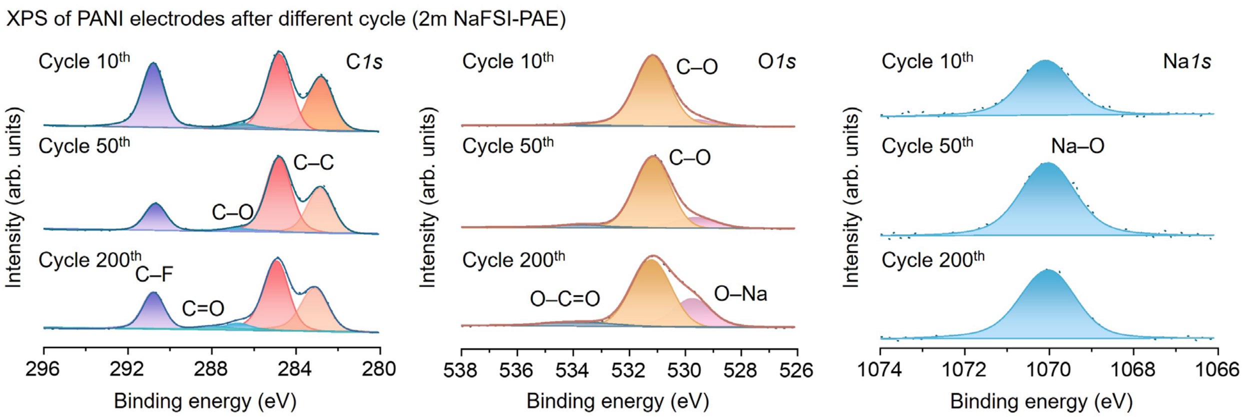

Supplementary Fig. 36. XPS spectra (C*1s*, O*1s*, and Na*1s*) of SEI on polyaniline anode after different cycles (10th, 50th, and 200th) in 2m NaFSI-PAE electrolyte.

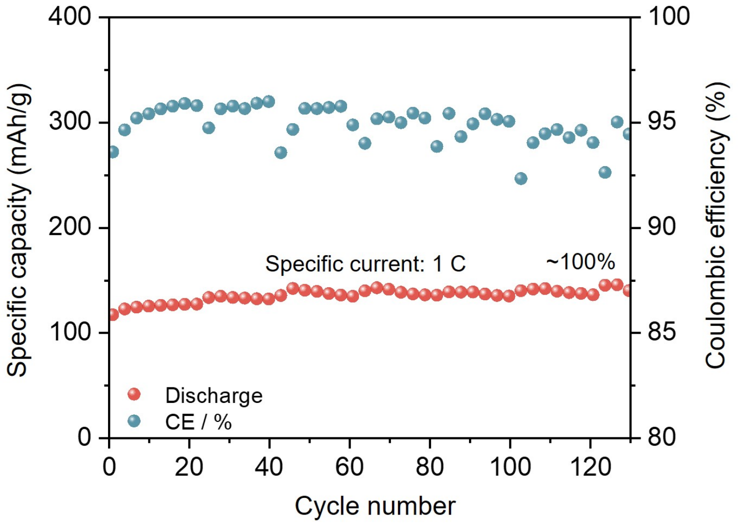

Supplementary Fig. 37. Cycling performance and coulombic efficiency of all-polymer films ASIBs.

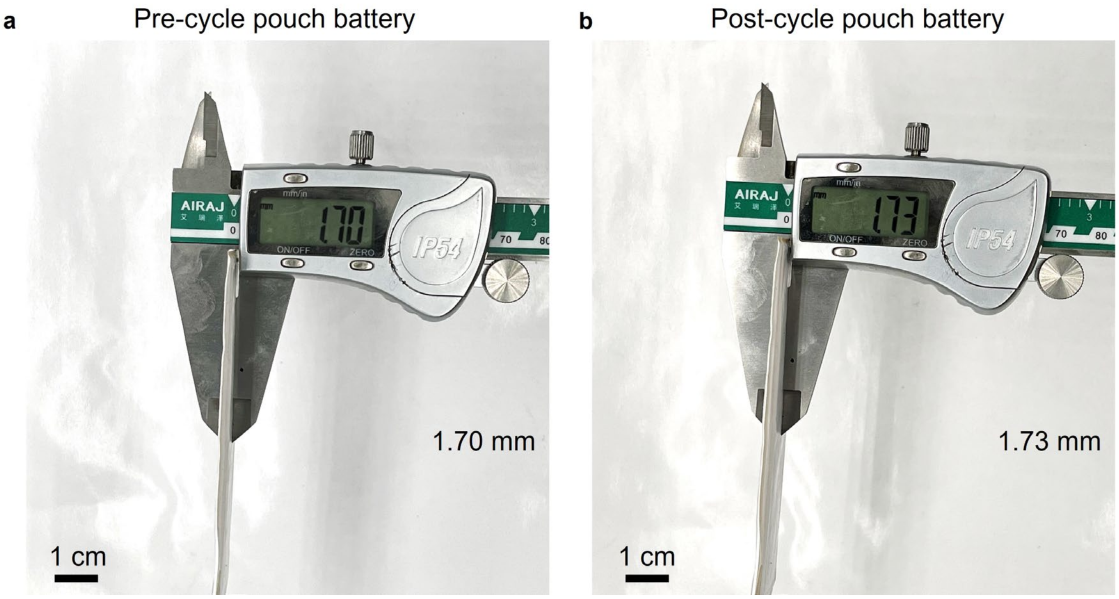

Supplementary Fig. 38. a, and b, Thickness of the pouch battery before and after charge-discharge cycling.

.

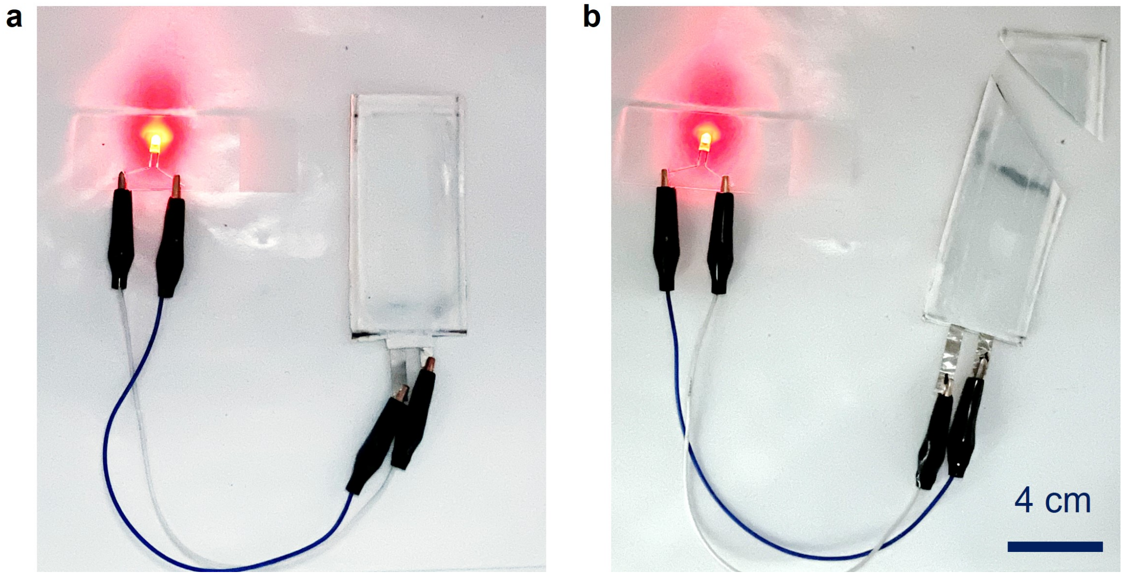

Supplementary Fig. 39. A digital photograph of a film battery to power an LED **a**, before and **b** after cutting.

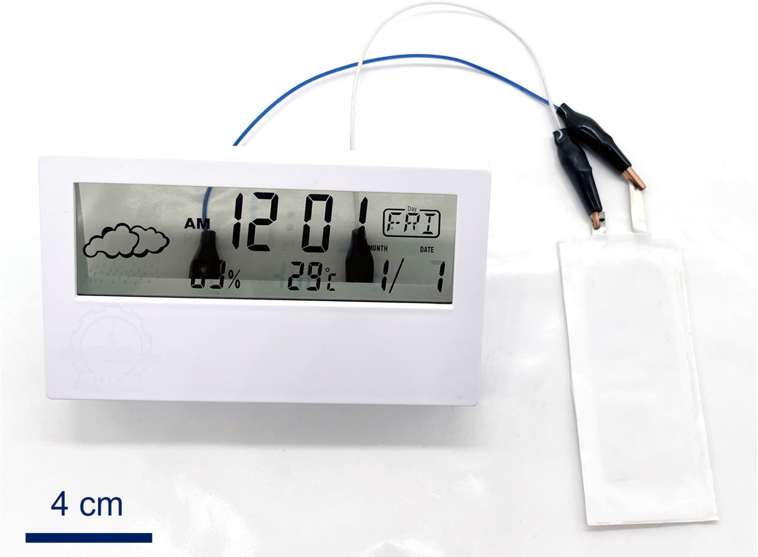

Supplementary Fig. 40. Digital photograph of a film battery to power a temperature, humidity recorder.

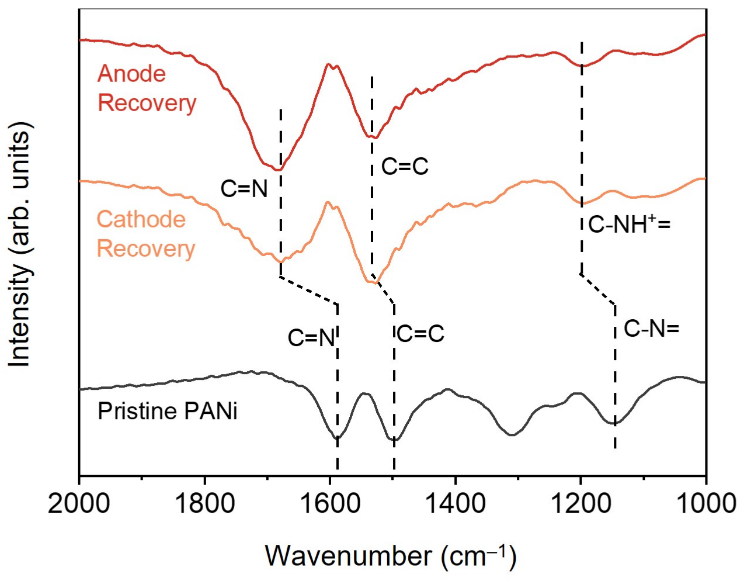

Supplementary Fig. 41. Comparison of FTIR of recycled cathode and anode with initial PANI.

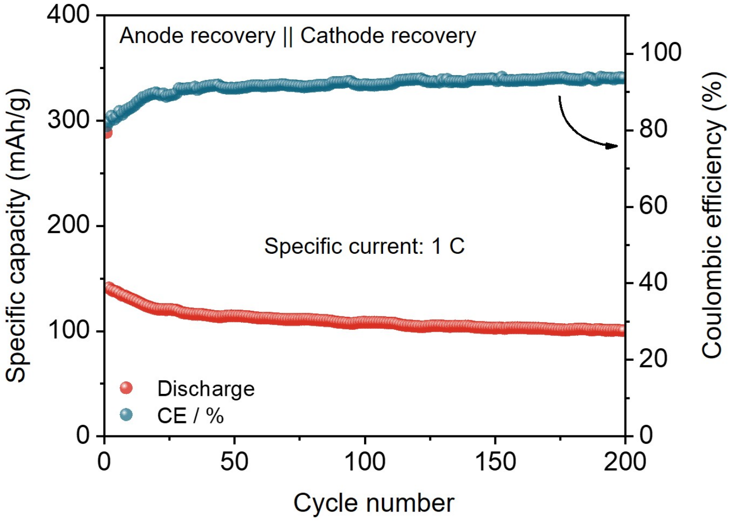

Supplementary Fig. 42. Cycling performance and coulombic efficiency of all-polymer ASIBs fabricated with recycled PANI.

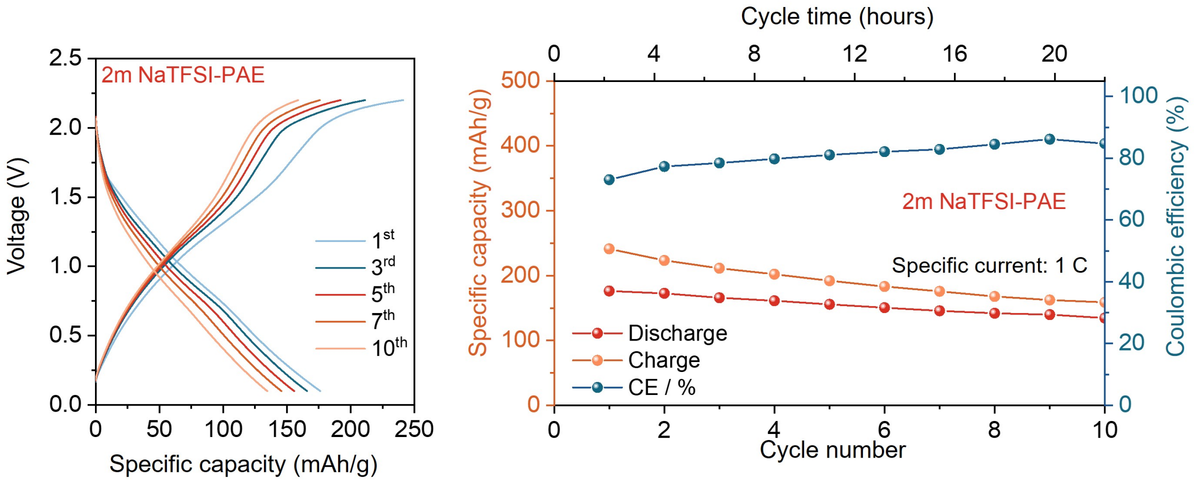

Supplementary Fig. 43. Electrochemical properties precycled for 10 cycles before cycling measurements. **a**, Galvanostatic charge-discharge curves of all-PANI battery at 1st, 3rd, 5th, 7th, and 10th. **b**, Cycling performance and coulombic efficiency in precycles.

Supplementary Table 1. Performance comparison table for ASIBs based on carboxyl derivates anodes and polyaniline cathodes.

**Materials**	**Specific capacity (mAh/g)**	**Cycle number**	**Capacity retention (%)**	**Average discharge voltage**	**Energy density (Wh/Kg)**
PTCDI‖PANI	56	400	66	1	56
NTCDA‖PANI	45	230	69	1	42
PNFE‖PANI	158	180	62	0.9	142
**This Work (PANI‖PANI)**	**139**	**4800**	**92**	**1.1**	**153**

The energy density was calculated based on the active materials mass of cathode electrodes.

Supplementary Table 2. FTIR spectra of PANI (emeraldine base).

Vibration	Wavenumber (cm^{-1})
δ (C–H)$_{oop}$	505 w
δ (C–N–C), δ (C–H)$_{oop}$	703 w
	831 m
δ (C–H)$_{ip}$, ν (C=N)	1171 s
	1302 s
ν (benzenoid rings, B-ring)	1492 s
ν (quinonoid rings, Q-ring)	1589 s

The "w", "m" and "s" refer to the intensity of the bands and stand for weak, medium, and strong, respectively. "oop" and "ip" refer to the bending vibration mode and stand for "out of plane" and "in plane" respectively.

Supplementary Table 3. FTIR spectra of PNFE.

Vibration	Wavenumber (cm^{-1})
ν^{as} (C–N–C)	766 s
ν (C–N)	1349 s
ν (naphthalene rings, N-ring)	1581 s
ν^{s} (C=O)	1669 s
ν^{as} (C=O)	1704 s

The “w”, “m” and “s” refer to the intensity of the bands and stand for weak, medium, and strong, respectively. “ν^{as}” and “ν^{s}” refer to asymmetric and symmetric stretching vibrations, respectively.

Supplementary Table 4. Comparison of reported ASIBs and aqueous Li-ion batteries with this work.

Type of battery	Materials	Specific capacity (mAh/g)	Energy density (Wh/Kg)	Cycle number	Capacity retention (%)	Average discharge voltage	Electrolyte concentration (mol/Kg)	Metal free	Ref
**Coin**	**PANI||PANI**	**139**	**153**	**4800**	**92%**	**1.1**	**2**	**1**	**This work**
Coin	($Na_2MnFe(CN)_6$|| $NaTi_2(PO_4)_3$	110	89	200<br>13000	~100%<br>74.3%	1.5	17	0	*Nat. Commun.* **15**, 575 (2024).[1]
Coin	PTCDA||AC	70	56	50	78%	0.8	2.3	1	*Angew. Chem. Int. Ed.* **60**, 24709–24715 (2021).[2]
Coin	PNFE||PTVE	75	131	200	100%	1.75	1	1	*Chem. Commun.* **51**, 5097–5099 (2015).[3]
Coin	NVP@C||NVP@C	40	77	100	87.5%	1.75	19	0	*Angew. Chem. Int. Ed.* **60**, 11943–11948 (2021).[4]
Coin	NaMnHCF|| NaTiO PO_4	70	122	200<br>800	90%<br>76%	1.7	31	0	*Adv. Mater.* **32**, 1904427 (2020).[5]
Coin	$Na_3V_2(PO_4)_2F_3$|| $NaTi_2(PO_4)_3$	-	-	-	-	1.72	1	0	*Angew. Chem. Int. Ed.* **58**, 14202–14207 (2019).[6]
Coin	$Na_{0.66}[Mn_{0.66}Ti_{0.34}]O_2$ ||$NaTi_2(PO_4)_3$	31	31	1200	92%	1	10.26	0	*Adv. Energy Mater.* **7**, 1701189 (2017).[7]
Coin	$LiMn_2O_4$||$Li_4Ti_5O_{12}$	50	180	800	68%	2.5	2	0	*ACS Energy Lett.* **7**, 123–130 (2022).[8]
Coin	$LiMn_2O_4$|| TiO_2 (C-TiO_2)	40	88	200	78%	2.2	13.8	0	*Adv. Mater.* **34**, 2207040 (2022).[9]
Coin	Mo_6S_8||$LiMn_2O_4$	92	174	50, 2000	88%,<br>70%	1.9	5	0	*Nat. Chem.* **13**, 1061–1069 (2021).[10]

Coin	$LiMn_2O_4$‖$Li_4Ti_5O_{12}$	44	110	300	67.4%	2.5	2	0	*Nat. Mater.* **19**, 1006–1011 (2020).[11]

The “m” represents molality (mol/kg). The energy density was calculated based on the active materials mass of electrodes.

Supplementary Table 5. Comparison of reported film/pouch Li/Na-ion with this work.

Type of battery	Materials	Specific capacity (mAh/g)	Energy density (Wh/Kg)	Cycle number	Capacity retention (%)	Average discharge voltage	Electrolyte concentration (mol/Kg)	Metal-free	Ref
**Planar**	**PANI||PANI**	**135**	**148.5**	**130**	**~100%**	**1.1**	**2**	**1**	**This work**
Planar	$LiMn_2O_4$||$Li_4Ti_5O_{12}$	61.1	152	1000	87%	2.5	4.5	0	*Nat. Energy* **7**, 186–193 (2022).[12]
Planar	$LiMn_2O_4$||CC-$LiTi_2(PO_4)_3$	110	165	500	72.5%	1.5	2	0	*Science* **379**, 488–493 (2023).[13]
Planar	$Na_{0.44}MnO_2$|| $NaTi_2(PO_4)_3$@C	43	55.9	1000	60%	1.3	1	0	*Chem* **3**, 348–362 (2017).[14]
Planar	$LiMn_2O_4$||$Li_4Ti_5O_{12}$	101	151.5	1500	75%	1.5	2	0	*Mater. Today Energy* **19**, 100570 (2021).[15]
Planar	$LiVPO_4F$||$LiVPO_4F$	55	141	4000	88%	2.4	25	0	*Adv. Mater.* **29**, 1701972 (2017).[16]
Planar	$LiTi_2(PO_4)_3$|| $Li_{1.1}Mn_2O_4$	39	63	100	72%	1.6	2	0	*Angew. Chem. Int. Ed.* **55**, 7474–7477 (2016).[17]
Planar	$LiMn_2O_4$|| $LiTi_2(PO_4)_3$	82	123	400	65%	1.5	4	0	*Adv. Mater.* **34**, 2105120 (2022).[18]
Planar	$LiTi_2(PO_4)_3$||AC	122	-	-	-	1	1	0.5	*Adv. Funct. Mater.* **32**, 2203081 (2022).[19]
Planar	Ni-doped $LiMn_2O_4$|| $NaTi_2(PO_4)_3$	40	60.2	2000	98%	1.4		0	*Adv. Mater. Interfaces* **10**, 2202035 (2023).[20]

The "m" represents molality (mol/kg). The energy density was calculated based on the active materials mass of electrodes.

Reference

1. Wu, H. *et al.* Alkaline-based aqueous sodium-ion batteries for large-scale energy storage. *Nat. Commun.* **15**, 575 (2024).
2. Karlsmo, M., Bouchal, R. & Johansson, P. High-Performant All-Organic Aqueous Sodium-Ion Batteries Enabled by PTCDA Electrodes and a Hybrid Na/Mg Electrolyte. *Angew. Chem. Int. Ed.* **60**, 24709–24715 (2021).
3. Deng, W., Shen, Y., Qian, J. & Yang, H. A polyimide anode with high capacity and superior cyclability for aqueous Na-ion batteries. *Chem. Commun.* **51**, 5097–5099 (2015).
4. Jin, T. *et al.* High-Energy Aqueous Sodium-Ion Batteries. *Angew. Chem. Int. Ed.* **60**, 11943–11948 (2021).
5. Jiang, L. *et al.* High-Voltage Aqueous Na-Ion Battery Enabled by Inert-Cation-Assisted Water-in-Salt Electrolyte. *Adv. Mater.* **32**, 1904427 (2020).
6. Zheng, Q. *et al.* Sodium- and Potassium-Hydrate Melts Containing Asymmetric Imide Anions for High-Voltage Aqueous Batteries. *Angew. Chem. Int. Ed.* **58**, 14202–14207 (2019).
7. Suo, L. *et al.* “Water-in-Salt” Electrolyte Makes Aqueous Sodium-Ion Battery Safe, Green, and Long-Lasting. *Adv. Energy Mater.* **7**, 1701189 (2017).
8. Dong, D., Xie, J., Liang, Z. & Lu, Y.-C. Tuning Intermolecular Interactions of Molecular Crowding Electrolyte for High-Performance Aqueous Batteries. *ACS Energy Lett.* **7**, 123–130 (2022).
9. Zhou, A. *et al.* An Electric-Field-Reinforced Hydrophobic Cationic Sieve Lowers the Concentration Threshold of Water-In-Salt Electrolytes. *Adv. Mater.* **34**, 2207040 (2022).
10. Yue, J. *et al.* Aqueous interphase formed by CO_2 brings electrolytes back to salt-in-water regime. *Nat. Chem.* **13**, 1061–1069 (2021).
11. Xie, J., Liang, Z. & Lu, Y.-C. Molecular crowding electrolytes for high-voltage aqueous batteries. *Nat. Mater.* **19**, 1006–1011 (2020).
12. Xu, J. *et al.* Aqueous electrolyte design for super-stable 2.5 V $LiMn_2O_4$ || $Li_4Ti_5O_{12}$ pouch cells. *Nat. Energy* **7**, 186–193 (2022).
13. Shen, Q. *et al.* Liquid metal-based soft, hermetic, and wireless-communicable seals for stretchable systems. *Science* **379**, 488–493 (2023).
14. Guo, Z. *et al.* Multi-functional Flexible Aqueous Sodium-Ion Batteries with High Safety. *Chem* **3**, 348–362 (2017).

15. Zhou, Y., Wang, Z. & Lu, Y.-C. Flexible aqueous lithium-ion batteries with ultrahigh areal capacity and long cycle life. *Mater. Today Energy* **19**, 100570 (2021).
16. Yang, C. *et al.* Flexible Aqueous Li-Ion Battery with High Energy and Power Densities. *Adv. Mater.* **29**, 1701972 (2017).
17. Dong, X., Chen, L., Su, X., Wang, Y. & Xia, Y. Flexible Aqueous Lithium-Ion Battery with High Safety and Large Volumetric Energy Density. *Angew. Chem. Int. Ed.* **55**, 7474–7477 (2016).
18. Ye, T. *et al.* A Tissue-Like Soft All-Hydrogel Battery. *Adv. Mater.* **34**, 2105120 (2022).
19. Hu, Y. *et al.* A "Two-in-One" Strategy for Flexible Aqueous Batteries Operated at −80 °C. *Adv. Funct. Mater.* **32**, 2203081 (2022).
20. Zhang, X. *et al.* Boosting Li-Ion Storage Capability of Self-Standing Ni-Doped $LiMn_2O_4$ Nanowall Arrays as Superior Cathodes for High-Performance Flexible Aqueous Rechargeable Li-Ions Batteries. *Adv. Mater. Interfaces* **10**, 2202035 (2023).
